# The association between early introduction of tiny tastings of solid foods and duration of breastfeeding

**DOI:** 10.1186/s13006-023-00544-6

**Published:** 2023-01-16

**Authors:** Jenny Stern, Eva-Lotta Funkquist, Maria Grandahl

**Affiliations:** 1 Department of Women’s and Children’s Health, Uppsala University, 751 85 Uppsala, Sweden; 2 Department of Health Promoting Science, Sophiahemmet University, 114 28 Stockholm, Sweden

**Keywords:** Breastfeeding, duration, Breastfeeding, exclusive, Infant, Mother, Solid foods

## Abstract

**Background:**

Conflicting advice and non-evidence-based recommendations have a negative effect on breastfeeding. Since 2011, the National Food Agency in Sweden has informed parents that they can introduce *tiny tastings* (1 mL of solid food, i.e. other sources of nutrition than breastmilk/formula) to infants from four months of age. It is unknown how national recommendations, which differ from the Word Health Organisation’s recommendation, affect breastfeeding. We hypothesised that introduction of tiny tastings of solid foods would shorten the duration of continued breastfeeding.

**Methods:**

This retrospective study utilises data from the longitudinal ‘Swedish Pregnancy Planning Study’, in which mothers were recruited at antenatal clinics on a national level. The participants completed three questionnaires up to one year after birth (*n* = 1,251). Linear regression models were used to analyse the association between the introduction of solid foods and the duration of breastfeeding.

**Results:**

As hypothesised, introduction of tiny tastings shortened the duration of continued breastfeeding. Half of all infants (48%) were fed with *tiny tastings* already in the fourth month. The correlation analysis showed that the earlier the infants started with *tiny tastings*, the earlier they ate larger amounts of solid food. In a multivariate linear regression analysis, five factors were identified as having a negative effect on the duration of breastfeeding: low infant age upon introduction of *tiny tastings*, low maternal age, low level of maternal education, high maternal BMI and twin birth.

**Conclusions:**

Early introduction of tiny tastings of solid foods shortened the duration of breastfeeding. It is difficult to influence most conditions that affect breastfeeding, for example, the mother’s educational level, BMI, age and if she has given birth to twins. In contrast, national guidelines can always be updated. Recommendations from the Swedish authorities should adhere to the WHO’s recommendation, which states exclusive breastfeeding for six months and continued breastfeeding for at least two years or longer.

**Supplementary Information:**

The online version contains supplementary material available at 10.1186/s13006-023-00544-6.

## Background

The Word Health Organisation (WHO) recommends exclusive breastfeeding for six months and continued breastfeeding for at least two years or longer [1, 2].

It is well known that infant formula feeding increases the risk of breastfeeding cessation [3]. However, research has shown conflicting results regarding whether early introduction of solid foods, i.e. other sources of nutrition than breastmilk/formula, is associated with a shorter duration of breastfeeding. In the Swedish study [3], no association was found, while the study from the UK showed both an association and a dose–response effect [4]. In this context, it is notable that these two countries differ greatly in breastfeeding habits. In a Norwegian study, early introduction of solid foods was related to maternal sociodemographic factors, such as lower educational level, smoking and lower age [5].

Even though breastfeeding is associated with health benefits for both the mother and the child, breastfeeding recommendations in several high-income countries are in conflict with the WHO’s recommendation [1]. It is unknown how national recommendations affect breastfeeding. Sweden is considered a pro-breastfeeding country, and the breastfeeding incidence peaked during the 1990s when more than 40% of infants were exclusively breastfed for six months [6]. This was partly a result of a planned strategy to increase breastfeeding rates, namely the Baby Friendly Hospital Initiative [7]. However, the prevalence of exclusive breastfeeding for six months has decreased over the last few decades to 11% in 2019 [6]. One recommendation, in particular, in the national guidelines, has possibly had a significant impact on breastfeeding. Since 2011, the National Food Agency has informed parents that they can introduce *tiny tastings* (1 mL of solid food) from four months of age. The reasoning was that the amount was so tiny that it would not negatively affect breastfeeding. In Sweden, all children aged 0–5 years are offered health care services free of charge by the Child Health Service (CHS). CHS is available in all the 290 communities and aims to contribute to children’s physical, psychological and social health by promoting health and development, preventing illness, detecting emerging problems early on, and intervening when needed to optimise the child’s development. Due to a non-evidence-based belief that exclusive breastfeeding might delay and complicate the introduction of solid foods, the CHS had not recommended exclusive breastfeeding for six months. Based on this misunderstanding, the National Food Agency changed the national guidelines and added the introduction of tiny tastings [8]. This change has been utilised by the Swedish baby food industry, offering a range of products intended for infants from four months of age. Furthermore, the CHS continues to encourage parents to start providing solid food at four months, even when women express that they want to breastfeed exclusively for six months [9]. In the present study, we hypothesised that early introduction of solid foods, including *tiny tastings,* was associated with shorter duration of breastfeeding.

## Methods

This retrospective study was a part of the longitudinal ‘Swedish Pregnancy Planning Study’ [10] and approved by the Swedish Ethical Review Authority, d.nr. 2010/085, with supplemental applications during the years. The aim was to investigate at what age solid foods, including *tiny tastings*, were introduced to Swedish infants as well as its effect on the duration of breastfeeding.

Antenatal clinics (*n* = 215) in ten (*n* = 10/21) regions in Sweden were invited to participate, and 153 (71%) agreed to participate. Women were asked to complete the questionnaires during registration at antenatal clinics in early pregnancy (Q1), in the third trimester (Q2) and one-year post-partum (Q3). The recruitment was conducted between 2012 and 2015. A total of 5,494 women were initially approached, out of which 4,969 women accepted participation. In the end, 3,389 completed and returned Q1. The first follow-up questionnaire (Q2) was sent to a total of 3,215 women, and 2,583 completed and returned the questionnaire. The second follow-up (Q3) was sent to 2,018 women, and 1,263 returned the questionnaire. A more detailed description of the procedure has been published previously [11]. Four of these questionnaires lacked an ID and could not be matched to previous questionnaires. For the current study, we also excluded women who had not provided data for all three questionnaires (*n* = 4), whose child was no longer alive (*n* = 3), and who provided data for an older sibling (*n* = 1). The final study sample comprises 1,251 women.

The self-reported questionnaires included sociodemographic questions about the mother (age, sex, previous children, country of birth, level of education and household income), the pregnancy (level of pregnancy planning, single/multiple pregnancy), mode of delivery (how it started, ended and if there was haemorrhage > 1000 ml), and the infant (birth weight, gestational age, sex, neonatal care, congenital states and twins). Furthermore, there was a detailed question about nutrition for the infant’s first year (0–12 months) “What kind of food did your child get during his/her first year?”: duration of breastfeeding, infant formula and introduction of solid foods including *tiny tastings*; see Additional file.

### Statistical analyses

The primary aim and sample characteristics were explored with descriptive statistics. Linear regression was used to analyse the effect of introducing solid foods (independent variable) on the duration of breastfeeding (dependent variable). Independent co-variables were chosen based on previous knowledge about breastfeeding and are presented in Table 1. Independent variables were analysed at the univariate level, and all significant variables were then included in the analysis at the multivariate level. Cox and Snell pseudo-R2 and Nagelkerke pseudo-R2 are presented as measures of the proportion of variation of outcomes explained by the model. For all statistical analyses, a two-sided *p*-value < 0.05 and a confidence interval (CI) of 95% were considered significant. Data were entered and analysed using IBM SPSS Statistics version 26 (IBM Corp. Armonk, NY, USA).

**Table 1:** Background characteristics of study population, *N* = 1251

*Characteristics of study sample*		*Study sample* *Mean (SD)*	*Study sample* *Frequency (%)*	*Comparison* *Official Statistics Sweden*
Mother	Age, years	29.9 (4.8)		30.3^a^
	Born outside Sweden		102 (8)	27.5^b^
	University education		701 (56)	49^b^
	Previous children		662 (53)	56^a^
	In a relationship		1221 (97)	34.0^a^
Pregnancy	Level of pregnancy planning			
	Highly planned		640 (51)	^d^
	Quite planned		339 (27)	^d^
	Neither planned nor unplanned		143 (11)	^d^
	Quite unplanned		38 (3)	^d^
	Highly unplanned		88 (7)	^d^
	Single pregnancy		1220 (98)	98^b^
	Multiple pregnancy		6 (0.5)	1.4^b^
Mode of delivery	Spontaneous vaginal		946 (76)	83^b^
	Induced vaginal		198 (16)	16.7^a^
	Planned Caesarean		98 (8)	8^b^
	Complications			
	Haemorrhage(> 1000 ml)		90 (7)	7.2^c^
	Emergency Caesarean		111 (9)	8^a^
	Instrumental delivery		99 (8)	7.2^a^
Infant	Birth weight, grams	3579 (548.8)		3565^b^
	Gestational age, weeks	40 (1.4)		39-40^b^
	Sex			
	Girl		612 (49)	48.6^b^
	Boy		636 (51)	51.3^b^
	Neonatal care		77 (6)	~ 10^b,e^
	Congenital state in need of care			
	Malformation		23 (2)	2-3^a^
	Injury		5 (0.4)	
	Disease		34 (3)	3.7/1000^a^

^**a**^ The National Board of Health and Welfare

^b^ Statistics Sweden

^c^ Vaginal (section = 10.4%)

^d^ No reliable data available

^e^ 5.5% gestation week < 37)

## Results

### The age at which solid foods were introduced

**Fig. 1:**
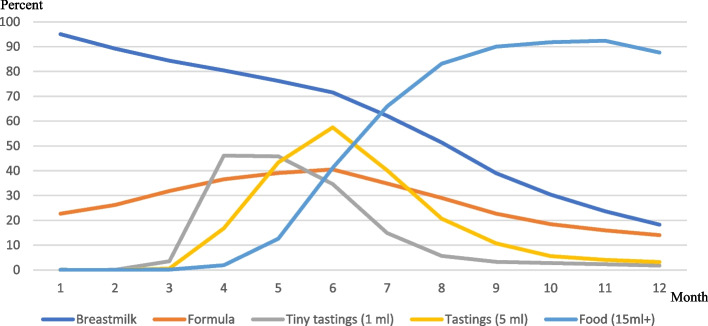
Infants’ nutrition during their first 12 months

Background characteristics of the mothers and their pregnancies, deliveries and infants are presented in Table 1. The Fig. 1 presents the infants’ nutrition intake during their first 12 months, including formula. The median age for introducing solid foods was during the fourth month. Almost all participants (94%) introduced their infant to solid foods during their fourth to seventh month, most commonly during their fourth month (48%). Tiny tastings (1 ml) were the most common kind of food intake during the third to fifth month, with tastings (5–10 ml) being the most common during the sixth month, and thereafter food in larger servings (15 ml or more); see Fig. 1. During their seventh month, more infants were fed with solid foods than with breastmilk. The regression analysis showed that the age when tiny tastings (1 ml) were introduced was associated with the age when food (15 ml or more) was introduced, i.e. the earlier the infants started with tiny tastings, the earlier they ate larger amounts of solid foods (β 0.813, *p* < 0.001).

### The effect of introduction of solid foods on the duration of breastfeeding

Variables associated with the duration of breastfeeding on univariate level were included in the multiple regression model and are presented in Table 2. In the multivariate linear regression analysis, higher maternal age, higher maternal education, lower maternal body mass index, singleton pregnancy and higher age at introduction of tiny tastings were associated with longer duration of breastfeeding (Table 2).

**Table 2:** Results for univariate and multivariate linear regression models with duration of breastfeeding during the first year after birth as the outcome

	**Univariate regression**						Multiple regression*			
**Variables**	***R Square***	***Adjusted R Square***	***Beta-coefficient***	***p***	**95,0% Confidence Interval for B**		***Beta-coefficient***	***p***	**95,0% Confidence Interval for B**	
Mother’s age	0.061	0.060	0.186	< 0.001	0.145	0.227	0.181	< 0.001*	0.130	0.232
Mother’s level of education	0.055	0.054	0.574	< 0.001	0.440	0.708	0.263	0.002*	0.099	0.426
Mother’s country of birth (outside *versus* inside Sweden)	0.004	0.003	0.821	< 0.001	0.083	1.559	0.279	0.496	-0.525	1.082
Household income	0.014	0.013	0.221	< 0.001	0.117	0.325	0.032	0.606	-0.090	0.154
Maternal leave during the first year	0.004	0.003	0.105	0.033	0.009	0.201	0.070	0.147	-0.025	0.166
Maternal Body Mass Index	0.035	0.034	-0.146	< 0.001	-0.190	-0.103	-0.129	< 0.001*	-0.177	-0.081
Pregnancy (single *versus* multiple)	0.010	0.009	-5.135	< 0.001	-8.027	-2.242	-4.057	0.032*	-7.768	-0.346
Mode of birth/end of delivery (normal *versus* instrumental/ caesarean)	0.005	0.004	-0.273	0.020	-0.502	-0.043	-0.120	0.322	-0.356	0.117
Age for introduction of solid foods (tiny tastings, tastings, or food)	0.044	0.043	0.703	< 0.001	0.520	0.886	0.590	< 0.001*	0.389	0.790

^*^ P < 0.05

^*^ Model summary R^2^ = 0.177 Adjusted R^2^ = 0.169

## Discussion

The main findings of this study were that half of all infants were fed with *tiny tastings* already in the fourth month and that the earlier the infant started with *tiny tastings*, the earlier they ate larger amounts of solid food. In the multivariate linear regression analysis, five factors were identified as having a negative effect on the duration of breastfeeding: the infant’s age upon introduction of *tiny tastings*, low maternal age, low level of maternal education, high maternal BMI and twin birth.

The Swedish recommendation to offer tiny tastings from four months of age contradicts the WHO’s recommendation [1], and its impact on breastfeeding has not been studied before. This study shows negative effects on breastfeeding duration. To recommend exclusive breastfeeding for six months could help to scale up breastfeeding and generate benefits besides those of the breastmilk itself, since breastmilk intake among children is associated with lower odds of consuming non-recommended foods, such as cookies, crackers and sweetened drinks [12, 13]. Instead, Nutrition Committees, such as the European Society for Paediatric Gastroenterology, Hepatology, and Nutrition, continue to emphasise the introduction of solid foods from four months of age [14]. In line with the recommendation, the Swedish CHS informs mothers that it is important to start with solid foods when the child is four months old, even when women express that they want to breastfeed exclusively for six months [9]. Addition of solid foods before six months is common in many countries, with one common argument being that this protects against developing food allergies. However, research has not found evidence of any benefits from addition of solid foods before six months, nor any risks related to morbidity or weight change [15]. Another common concern about exclusive breastfeeding for six months is the risk for iron-deficiency anaemia. However, the risk can be successfully mitigated by delayed umbilical-cord clamping [16].

Conflicting advice and non-evidence-based recommendations have a negative effect on breastfeeding [6]. In many ways, it can be perplexing for women to breastfeed in a society that is not infused by a favourable attitude towards breastfeeding. Consequently, breastmilk substitutes have become a “multi-billion dollar industry” that has the opportunity to devote considerable financial resources, influencing women not to breastfeed [17]. Thus, our findings are consistent with previous literature.

### Socioeconomic factors with an impact on breastfeeding

The multivariate linear regression analysis showed that low maternal age and low education had a negative impact on breastfeeding. In previous studies, several socioeconomic factors have shown an association with a shorter duration of breastfeeding. For example, mothers with less privileged economic situation and less education have a shorter duration of breastfeeding [18, 19], and mothers with lower age breastfeed for fewer months [19, 20]. This contributes to unequal starting points for children, already from birth. Thus, breastfeeding should be the focus of targeted interventions from the CHS in order to promote equal health. The United Nations’ Sustainable Development Goals compel governments to promote healthy lives and welfare for all. As discussed in previous research, this makes breastfeeding a central part of the 2030 Agenda, since it contributes to the achievement of an equal, healthy, fair, affluent and sustainable future for both people and the planet [21].

### Obesity and breastfeeding

This study showed that high BMI in the mother was a significant factor for the shorter duration of breastfeeding. Maternal obesity is linked to many risks, with one of them being a lower initiation rate of breastfeeding and also a greater risk of early breastfeeding cessation [22, 23]. It has been suggested that the causes can be a mix of physiological, behavioural, sociocultural, psychological and medical reasons. For example, obese women can have higher progesterone levels, which may impair lacto genesis. Furthermore, large breasts may lead to problems for the infant to latch on, and the obese mother may lack confidence in the breastfeeding situation because of low body image [24].

### Less breastfeeding among twins

Another factor identified as having a negative effect on the duration of breastfeeding in the multivariate linear regression analysis was twin births. Women who have given birth to twins face special challenges, and breastfeeding rates are lower among these infants [25]. According to the mothers, the reasons for weaning twins are insufficient milk supply and infants’ problematic breastfeeding behaviour [26]. This indicates that mothers of twins need targeted breastfeeding support that takes into account these mothers’ unique situation.

### Strengths and limitations

This study investigated the impact of tiny tastings on breastfeeding. Data were obtained from many mothers (*n* = 1,260), and the sample represents different geographical areas, including both high and low socioeconomic statuses. The question measuring breastfeeding duration (exclusive and partial) is very detailed; consequently, it may be more reliable than the Swedish national data [6], even though the retrospective data is a limitation, due to potential recall bias. Conversely, the study design cannot provide causes; rather, it shows associations. The response rate for the follow-up (Q3, *n* = 1,251), compared with baseline data (a total of 3,389 women completed and returned Q1), was lower. In addition, there might be selection bias, since the study design excluded non-Swedish speaking parents. Moreover, the study’s findings cannot be generalised to infants in other countries than Sweden.

## Conclusion

This study investigated the impact of *tiny tastings* on the duration of breastfeeding*.* The results revealed that half of all Swedish infants taste their first solid foods during their fourth month, and that the earlier the tiny tastings were introduced, the shorter the duration of breastfeeding. It is difficult to influence most conditions that affect breastfeeding, for example, the mother’s educational level, BMI, age and if she has given birth to twins. In contrast, national guidelines can always be updated. Recommendations from the Swedish authorities should adhere to the WHO’s recommendation, which states exclusive breastfeeding for six months and partial breastfeeding for at least two years or longer [1].

## Supplementary Information

**Additional file 1.** Surveykey question. Item measuring the child’s food.

## Data Availability

The datasets used and/or analysed during the current study are available from the corresponding author upon reasonable request.

## Abbreviations

BMI: Body Mass Index
Q1, Q2, Q3: Questionnaire 1, Questionnaire 2, Questionnaire 3
WHO: World Health Organisation

## References

[1] World Health Organization. Health Topics, Breastfeeding. 2018. https://www.who.int/health-topics/breastfeeding#tab=tab_2. Accessed 12 Dec 2021.

[2] Kramer MS, Kakuma R (2012). Optimal duration of exclusive breastfeeding. Cochrane Database Syst Rev..

[3] Hornell A, Hofvander Y, Kylberg E (2001). Solids and formula: association with pattern and duration of breastfeeding. Pediatrics.

[4] Lessa A, Garcia AL, Emmett P, Crozier S, Robinson S, Godfrey KM (2020). Does early introduction of solid feeding lead to early cessation of breastfeeding?. Matern Child Nutr..

[5] Helle C, Hillesund ER, Overby NC (2018). Timing of complementary feeding and associations with maternal and infant characteristics: a Norwegian cross-sectional study. PloS One.

[6] The National Board of Health and Welfare. Statistics on breastfeeding, 2019. https://www.socialstyrelsen.se/statistik-och-data/statistik/statistikamnen/amning/. Accessed 16 Dec 2021.

[7] Hofvander Y (2005). Breastfeeding and the Baby Friendly Hospitals Initiative (BFHI): organization, response and outcome in Sweden and other countries. Acta Paediatr.

[8] Swedish National Food Agency. Råd om mat för barn 0–5 år. Hanteringsrapport som beskriver hur risk och nyttovärderingar, tillsammans med andra faktorer, har lett fram till Livsmedelsverkets råd. In English: [Advice on food for children 0–5 years. Management report that describes how risk and utility valuations, together with other factors, have led to the Swedish National Food Agency's advice.] 2011. https://www.livsmedelsverket.se/globalassets/publikationsdatabas/rapporter/2011/2011_livsmedelsverket_22_rad_om_mat_barn_0_till_5_hanteringsrapport.pdf.

[9] Blixt I, Johansson M, Hildingsson I, Papoutsi Z, Rubertsson C (2019). Women's advice to healthcare professionals regarding breastfeeding: "offer sensitive individualized breastfeeding support"- an interview study. Int Breastfeed J.

[10] Stern J, Salih Joelsson L, Tyden T, Berglund A, Ekstrand M, Hegaard H (2016). Is pregnancy planning associated with background characteristics and pregnancy-planning behavior?. Acta Obstet Gynecol Scand.

[11] Grandahl M, Stern J, Funkquist EL (2020). Longer shared parental leave is associated with longer duration of breastfeeding: a cross-sectional study among Swedish mothers and their partners. BMC Pediatr.

[12] Spaniol AM, da Costa THM, Bortolini GA, Gubert MB (2020). Breastfeeding reduces ultra-processed foods and sweetened beverages consumption among children under two years old. BMC Public Health.

[13] Passanha A, Benicio MHD, Venancio SI (2018). Influence of breastfeeding on consumption of sweetened beverages or foods. Rev Paul Pediatr.

[14] Fewtrell M, Bronsky J, Campoy C, Domellof M, Embleton N, Fidler Mis N (2017). Complementary feeding: a position paper by the European Society for Paediatric Gastroenterology, Hepatology, and Nutrition (ESPGHAN) committee on nutrition. J Pediat Gastroenterol Nutr.

[15] Smith HA, Becker GE (2016). Early additional food and fluids for healthy breastfed full-term infants. Cochrane Database Syst Rev.

[16] Perez-Escamilla R, Buccini GS, Segura-Perez S, Piwoz E (2019). Perspective: should exclusive breastfeeding still be recommended for 6 months?. Adv Nutr.

[17] Rollins NC, Bhandari N, Hajeebhoy N, Horton S, Lutter CK, Martines JC (2016). Why invest, and what it will take to improve breastfeeding practices?. Lancet.

[18] Bjorset VK, Helle C, Hillesund ER, Overby NC (2018). Socio-economic status and maternal BMI are associated with duration of breast-feeding of Norwegian infants. Public Health Nutr.

[19] Smith HA, O’B Hourihane J, Kenny LC, Kiely M, Murray DM, Leahy-Warren P (2015). Early life factors associated with the exclusivity and duration of breast feeding in an Irish birth cohort study. Midwifery.

[20] Pruszkowska-Przybylska P, Sitek A, Rosset I, Zadzinska E, Sobalska-Kwapis M, Slomka M (2019). The association between socioeconomic status, duration of breastfeeding, parental age and birth parameters with BMI, body fat and muscle mass among prepubertal children in Poland. Anthropol Anz.

[21] Unicef. Breastfeeding and the Sustainable Development Goals. 2016. https://worldbreastfeedingweek.org/2016/pdf/BreastfeedingandSDGsMessaging%20WBW2016%20Shared.pdf. Accessed 3 Jan 2022.

[22] Bautista-Castano I, Henriquez-Sanchez P, Aleman-Perez N, Garcia-Salvador JJ, Gonzalez-Quesada A, Garcia-Hernandez JA (2013). Maternal obesity in early pregnancy and risk of adverse outcomes. PloS One.

[23] Fan WQ, Molinaro A (2021). Maternal obesity adversely affects early breastfeeding in a multicultural, multi-socioeconomic Melbourne community. Aust N Z J Obstet Gynaecol.

[24] Marchi J, Berg M, Dencker A, Olander EK, Begley C (2015). Risks associated with obesity in pregnancy, for the mother and baby: a systematic review of reviews. Obes Rev.

[25] Ostlund A, Nordstrom M, Dykes F, Flacking R (2010). Breastfeeding in preterm and term twins–maternal factors associated with early cessation: a population-based study. J Hum Lact..

[26] Mikami FCF, Francisco RPV, Rodrigues A, Hernandez WR, Zugaib M, de Lourdes BM (2018). Breastfeeding Twins: Factors Related to Weaning. J Hum Lact.

