# Supplementary material for: The association between early introduction of tiny tastings of solid foods and duration of breastfeeding
Source: Int Breastfeed J. 2023 Jan 16;18:4. doi: 10.1186/s13006-023-00544-6 (PMC9843836; doi:10.1186/s13006-023-00544-6)
Supplement: Supplementary file 1 — Additional file 1. Surveykey question. Item measuring the child’s food. [file 13006_2023_544_MOESM1_ESM.docx]

Supplemental file.

Survey key question. Item measuring the child’s food

What kind of food did your child get during his/her first year? Tick all options that applies to your child for each month. Several options can be ticked for each month.

Select all options that applies for each month:
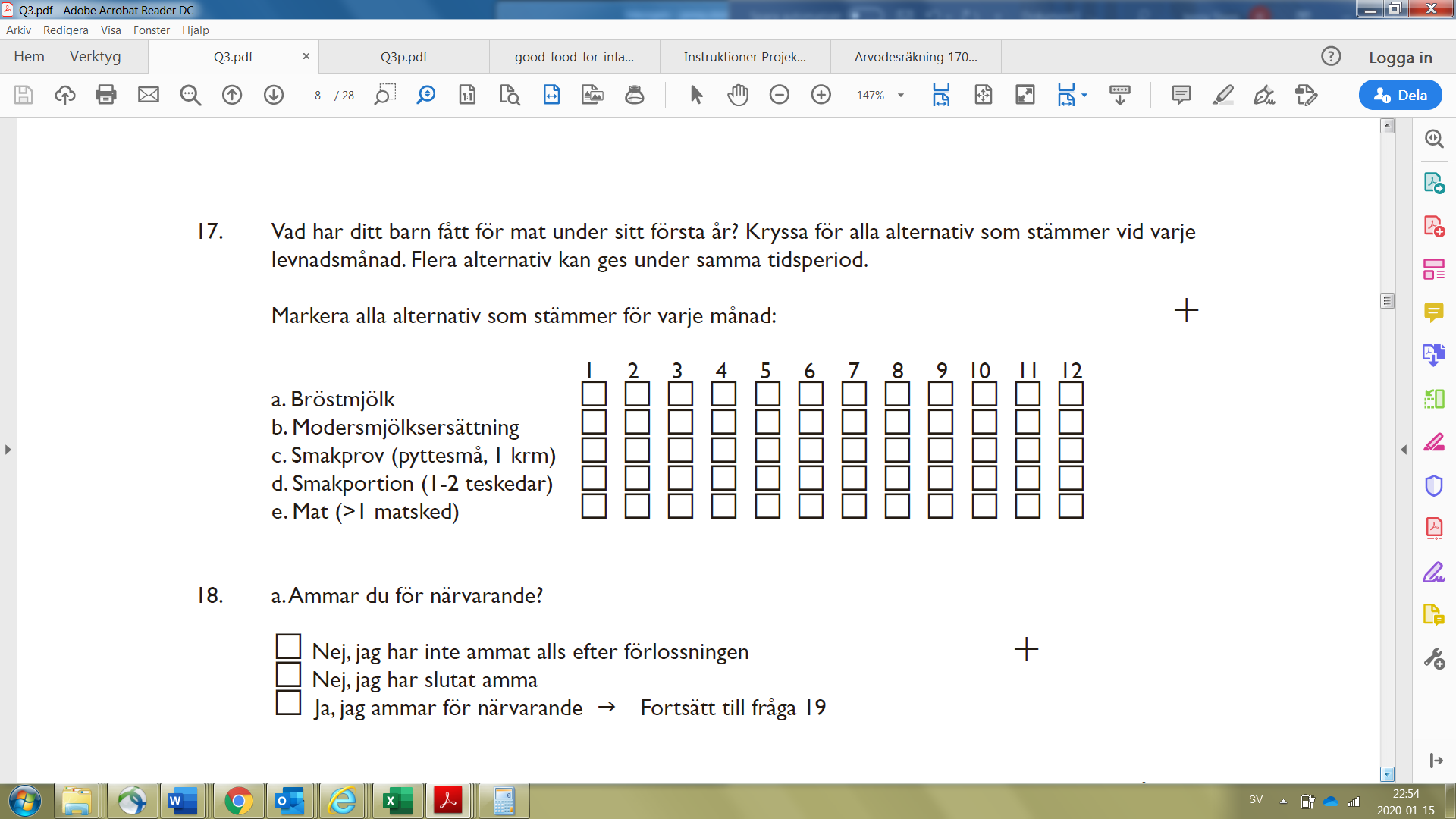


1. Breast milk
2. Infant formula
3. Tiny little taste (tiny tastings, 1 mL)
4. Small samples (1-2 teaspoons)
5. Food (> 1 tablespoons)

Swedish:

Vad har ditt barn fått för mat under sitt första år? Kryssa för alla alternativ som stämmer vid varje levnadsmånad. Flera alternativ kan ges under samma tidsperiod.


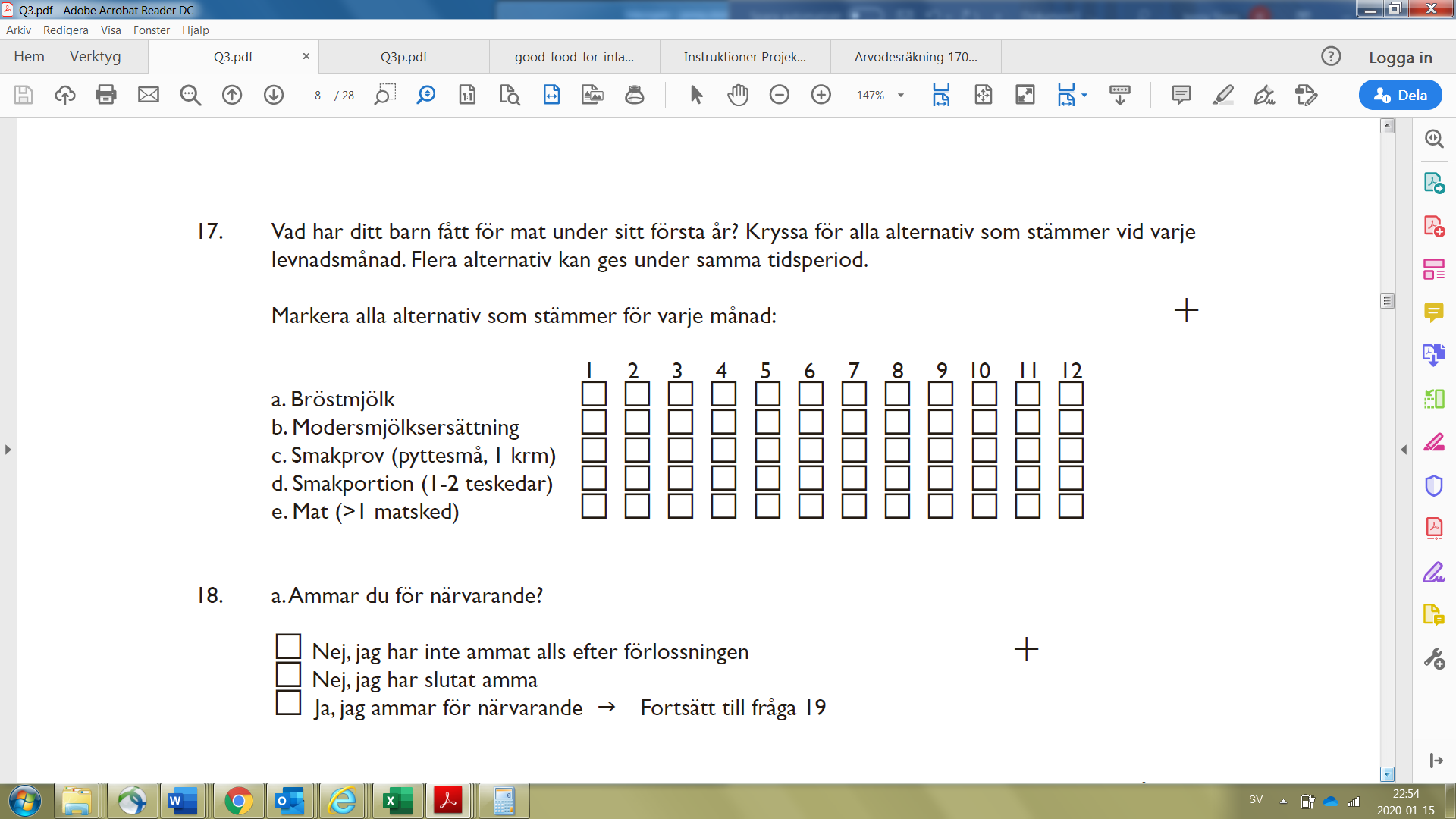
Markera alla alternativ som stämmer för varje månad:

1. Bröstmjölk
2. Modersmjölksersättning
3. Smakprov (pyttesmå, 1 krm)
4. Smakportion (1-2 teskedar)
5. Mat (>1 matsked)
